# STRIKE-HBV: establishing an HBV screening programme in Kilifi, Kenya—challenges, successes and lessons learnt

**DOI:** 10.1136/sextrans-2024-056163

**Published:** 2024-05-24

**Authors:** Louise O Downs, Oscar Chirro, Mwanakombo Zaharani, Benson Safari, Dorcas Okanda, George Githinji, Monique I Andersson, Rob Newton, Anthony Etyang, Nadia Aliyan, Philippa Clare Matthews

**Affiliations:** 1 Nuffield Department of Medicine, University of Oxford, Oxford, UK; 2 KEMRI-Wellcome Trust Research Programme, Kilifi, Kenya; 3 Department of Biochemistry and Biotechnology, Pwani University, Kilifi, Kenya; 4 Microbiology and Infectious Diseases, Oxford University Hospitals NHS Foundation Trust, Oxford, UK; 5 Radcliffe Department of Medicine, University of Oxford, Oxford, UK; 6 Uganda Virus Research Institute, Entebbe, Wakiso, Uganda; 7 Department of Health Sciences, University of York, York, UK; 8 Epidemiology and Demography, KEMRI-Wellcome Trust Research Programme, Kilifi, Kenya; 9 Kilifi District Hospital, Kilifi, Kenya; 10 The Francis Crick Institute, London, UK; 11 Division of Infection and Immunity, University College London, London, UK

**Keywords:** HEPATITIS B, VIROLOGY, Implementation Science, PUBLIC HEALTH

## Abstract

**Objectives:**

Chronic hepatitis B infection affects 65 million people in the WHO African Region, but only 4.2% of these are diagnosed and 0.2% on treatment. Here, we present a short report describing establishment of a hepatitis B virus (HBV) programme in Kenya. We share experiences, successes and challenges to support development of future programmes.

**Methods:**

From March 2023, we began the ‘STRIKE-HBV’ Study to identify people living with HBV (PLWHB) in Kilifi, Kenya. We employed local staff and provided education and training. Individuals were identified through three routes: (1) we offered free-of-charge HBV testing for all non-pregnant adults attending Kilifi Country Hospital (KCH) outpatient department; (2) we invited PLWHB to reattend for review; and (3) we invited close contacts of PLWHB for screening and vaccination if HBV was negative. All those seropositive for HBV were offered a comprehensive liver health assessment.

**Results:**

We have established a framework for HBV screening, assessment and linkage to care in Kilifi. Between March 2023 and March 2024, we collected data for 80 PLWHB, comprising (1) screening of 1862 people of whom 30 were seropositive, (2) enrolment of 38 people known to be living with HBV and (3) testing of 97 close contacts of PLWHB, of whom 12 were positive. Among a limited subset with elastography data, we identified 9 of 59 as having significant fibrosis, and a further 6 people had laboratory aspartate transaminase (AST) to platelet ratio index (APRI) scores in keeping with fibrosis. We encountered challenges including procurement delays for hepatitis B surface antigen testing kits and HBV vaccinations, and issues accessing liver elastography.

**Conclusions:**

HBV screening was well received by the Kilifi population, has identified people at risk of liver disease progression and is improving linkage to care and vaccination at KCH. Future HBV programmes in WHO Africa can build on this experience as we work to develop accessible, affordable and acceptable care pathways.

**WHAT IS ALREADY KNOWN ON THIS TOPIC**

- Screening for hepatitis B virus (HBV) infection, vaccination for household/sexual contacts and clinical care for those with chronic HBV disease are not available in much of the WHO African Region.

**WHAT THIS STUDY ADDS**

- We describe our experience of establishing HBV screening in a county hospital in Kenya, to provide accessible, affordable and acceptable access to HBV services.

**HOW THIS STUDY MIGHT AFFECT RESEARCH, PRACTICE OR POLICY**

- We have shown that with a robust programme of education, both the local population and healthcare workers are keen to engage with HBV testing. We have outlined mechanisms by which HBV clinical care can be strengthened and advocate that improvements in HBV care should be a priority for Kenya.

## Introduction

Approximately 65 million people in the WHO African Region (WHO AFRO) are living with chronic hepatitis B infection (CHB)^1^; however, only around 4.2% of these people are diagnosed and 0.2% are on treatment.^2^ There is therefore an urgent need to scale up hepatitis B virus (HBV) testing programmes throughout the WHO AFRO.

We here focus on a population in Kilifi County on the Kenyan coast. The estimated CHB prevalence in Kenya is 3–5%, although this is based on data from unrepresentative populations^3^ and may vary significantly by region. In Kenya, HBV testing is not routine or free in many government hospitals, and HBV vaccination was only introduced into the infant expanded programme for immunisation in 2001.^4^ Lack of access to HBV education, prevention, diagnostics and treatment results in a population at risk of individual liver complications and maintains a population reservoir for continued transmission.

Although Kenyan guidelines on the management of CHB currently advocate HBV biomarker measurement including HBV DNA and hepatitis B ‘e’ antigen,^5^ there is no mechanism to assess viral replication or liver health other than blood tests such as a complete blood count and alanine aminotransferase (ALT) in most settings, and pathways to care are not well established. At Kilifi County Hospital (KCH), people living with HBV (PLWHB) have been offered nucleos(t)ide analogue (NA) therapy free of charge, usually tenofovir/lamivudine combination therapy, which is accessible through the national HIV treatment programme. Updated WHO guidelines published in March 2024 endorse this dual therapy approach and the inclusive approach to treatment in the absence of access to detailed stratification.^6^

In this short report, we outline the implementation of an opt-in HBV screening and clinical assessment programme through KEMRI-Wellcome Trust Research Programme at KCH (the ‘STRIKE-HBV Study’), aiming to describe the implementation framework used, and share challenges and successes along with preliminary results to support the development of future similar community initiatives to tackle HBV infection.

## Methods

### Study location and timeline

KCH is a referral hospital servicing an area of 12 000 km^2^ and a population of 1.5 million people. KCH has 300 inpatient beds and sees 193 000 outpatients every year. A small number of PLWHB are currently managed at KCH through the HIV service, but prior to STRIKE-HBV, there has been no active mechanism for case finding, screening of contacts or vaccination. From March 2023, we began recruiting adults living with HBV infection into a clinical research programme. Data were collected using a REDCap database.^7^

### Education, training and consent

We presented STRIKE-HBV to the hospital, subcounty and county management teams for sensitisation and approval, and then engaged with the KCH medical team to facilitate a testing space. We employed and trained study staff and conducted hospital healthcare worker (HCW) sensitisation sessions.

**Figure 1:**
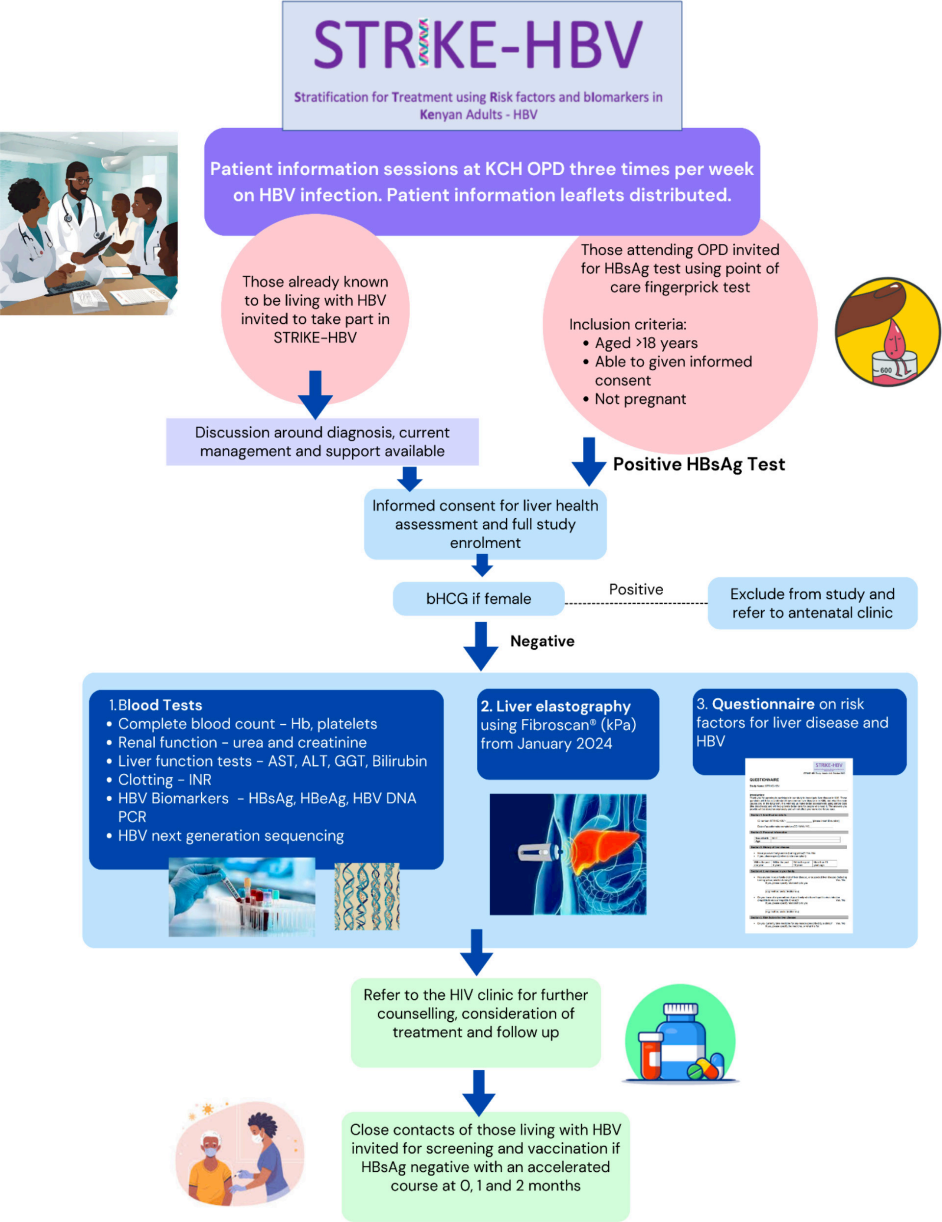
Flow of the STRIKE-HBV Study—a cross-sectional study recruiting outpatients attending Kilifi County Hospital (KCH), Kenya to undertake hepatitis B surface antigen (HBsAg) testing, liver health assessment, referral to clinical care, tracing and vaccination of close contacts. Inclusion criteria are shown along with investigations undertaken on those testing HBsAg positive, referral and vaccination pathways. ALT, alanine aminotransferase; AST, aspartate aminotransferase; bHCG, beta-human chorionic gonadotropin; GGT, gamma-glutamyl transpeptidase; Hb, haemoglobin; HBeAg, hepatitis B ‘e’ antigen; HBV, hepatitis B virus; INR, international normalised ratio; kPa, kilopascals; OPD, outpatient department.

Working with the Hepatitis B Foundation,^8^ we developed educational resources in English and Kiswahili (https://doi.org/10.6084/m9.figshare.c.7114813.v2) to support information sessions led by fieldworkers in outpatient waiting areas during screening clinics. The flow of the study is shown in figure 1. We undertook group consent for HBV screening supported by individual discussions if needed. For those known to be living with HBV and managed at KCH, the HIV team invited them to an HBV information session and offered the opportunity to join the study.

### Recruitment pathways

PLWHB were identified through three routes:

1. We established voluntary free-of-charge HBV screening at KCH outpatient department (OPD), initially once weekly, but increasing to 3 days/week.
2. We invited those known to be living with HBV to attend for reassessment, supported by local HIV services as above.
3. We invited PLWHB to bring their close contacts for hepatitis B surface antigen (HBsAg) testing. We provided liver health assessment for anyone testing HBsAg positive and vaccinated those HBsAg negative.

### HBsAg screening

Testing was done using a point-of-care (POC) HBsAg fingerprick test (first using QuickProfile HBsAg kits (LumiQuick Diagnostics, California, USA) and subsequently determine HBsAg 2 (Abbott, USA)). Anyone testing positive for HBsAg had their results discussed privately and provided consent for additional liver health assessment.

### Liver health assessment

All PLWHB enrolled in STRIKE-HBV were offered blood tests and a lifestyle questionnaire (https://doi.org/10.6084/m9.figshare.25714347.v1). Liver stiffness assessment was undertaken using Fibroscan Mini+ 430 (Echosens, Paris). We calculated APRI scores using the formula: $\frac{AST/AST\left(ULN\right)}{Platelets}x100$ . Aspartate aminotransferase (AST) upper limit of normal (ULN) was taken as 42 IU/L for women and 47 IU/L for men as per local Kilifi reference ranges. Elastography scores >7 kPa and APRI scores >0.5 were considered significant fibrosis. All quantitative analyses were performed using Microsoft Excel V.16.8.

### Funding

This project is funded by the Wellcome Trust (grant 225485/Z/22/Z) and the John Fell Fund, University of Oxford (ref: 0012112).

## Results

### Screening numbers and characteristics

Between March 2023 and March 2024 inclusive, we screened 1862 people for HBV (75% female, median age 41 years, range 18–98 years) and we scaled up testing as the study progressed (online supplemental figures 1 and 2). 30 people were newly tested HBsAg positive (17 female, 13 male, median age 45 years, range 21–86 years). We also enrolled 38 people already living with HBV (25 female, 13 male, median age 34 years, range 25–60 years) and tested 97 household or sexual contacts for HBsAg of whom 12 were also positive (12.4%, 7 female, 5 male, median age 38 years, range 25–55 years).

10.1136/sextrans-2024-056163.supp1 Supplementary data

10.1136/sextrans-2024-056163.supp2 Supplementary data

### Liver health assessment

Elastography scores were available for 27 participants newly diagnosed with HBV and 32 participants already known to be living with HBV. Five of 27 and 4 of 32 in each group, respectively, had significant fibrosis based on elastography (prevalence 9 of 59, 15.3%) and a further 6 participants were identified based on APRI score (prevalence 15 of 59, 25.4%).

### Positive outcomes of screening

STRIKE-HBV has created awareness around HBV both in the Kilifi population and HCWs at KCH. Often community members attend the clinic based on personal recommendation, and attended to seek information, support and referral for care. Five education sessions have been held for hospital staff and have been well received (https://doi.org/10.6084/m9.figshare.25526284.v1). As a result of this work, KCH is in the process of undertaking free HBV vaccination for all HCWs (https://doi.org/10.6084/m9.figshare.25718268) and the current HBV clinical care service is developing a peer support network for PLWHB.

### Challenges initiating the programme

- *Procurement issues:* we used a combination of HBsAg testing kits because of significant delays due to central and local distributor issues, manufacturing and importation time.
- *Elastography measurement:* this was delayed while negotiating access to hardware. Purchasing a new or secondhand machine was prohibitively expensive, so we organised a loan machine which only became available 10 months after study initiation.
- *Vaccination:* obtaining monovalent HBV vaccination was challenging due to national stock issues and then delivery without temperature monitoring. Eventually, single-dose vaccine vials were obtained, but at significantly higher cost than multidose vials.
- *Screening capacity:* scale-up was limited by location and availability of clinic space and number of staff.

## Discussion

Our approach to establishing an HBV programme in a county hospital in Kenya demonstrates that with appropriate support and education, screening for HBV and referral to clinical care are feasible and acceptable and identify people with liver disease requiring NA therapy to prevent further disease progression. A high proportion of the people we tested were women due to the demographic of those attending KCH. The lower engagement of men is concerning given their higher risk of HBV and related liver complications.^9 10^ Expansion of testing into communities may improve screening coverage, but also education targeting men specifically may be needed.

HBsAg POC tests well validated in African populations^8^ are expensive and difficult to access, sometimes meaning less well-validated tests are used. Elastography machines are not easily available; however, recent WHO guidelines advocate the use of APRI scores.^6^ Vaccine access was consistently challenging, highlighting the need for government and global commitment to ensure consistent, equitable supply of vaccines.

When project recruitment is complete, we will undertake standardisation of data in our OPD population for age and sex against the Kilifi population structure to enable HBV prevalence estimation. Cost-effectiveness of such programmes requires careful evaluation, considering the short-term resource implications of scale-up, offset by long-term gains in population health. Simplified WHO guidelines^6^ reduce the cost implications of stratification for treatment, setting the scene for a scale-up of interventions.

## Conclusions

HBV screening in KCH was well received and identified people with liver disease. People already diagnosed but not engaged in care were linked into the HIV clinic for ongoing management. Scale-up of this programme is needed to allow increased screening availability while balancing affordability and acceptability.
