# Supplementary material for: STRIKE-HBV: establishing an HBV screening programme in Kilifi, Kenya—challenges, successes and lessons learnt
Source: Sex Transm Infect. 2024 May 24;100(5):325–8. doi: 10.1136/sextrans-2024-056163 (PMC11287631; doi:10.1136/sextrans-2024-056163)
Supplement: Supplementary data [file sextrans-2024-056163supp001.pdf]

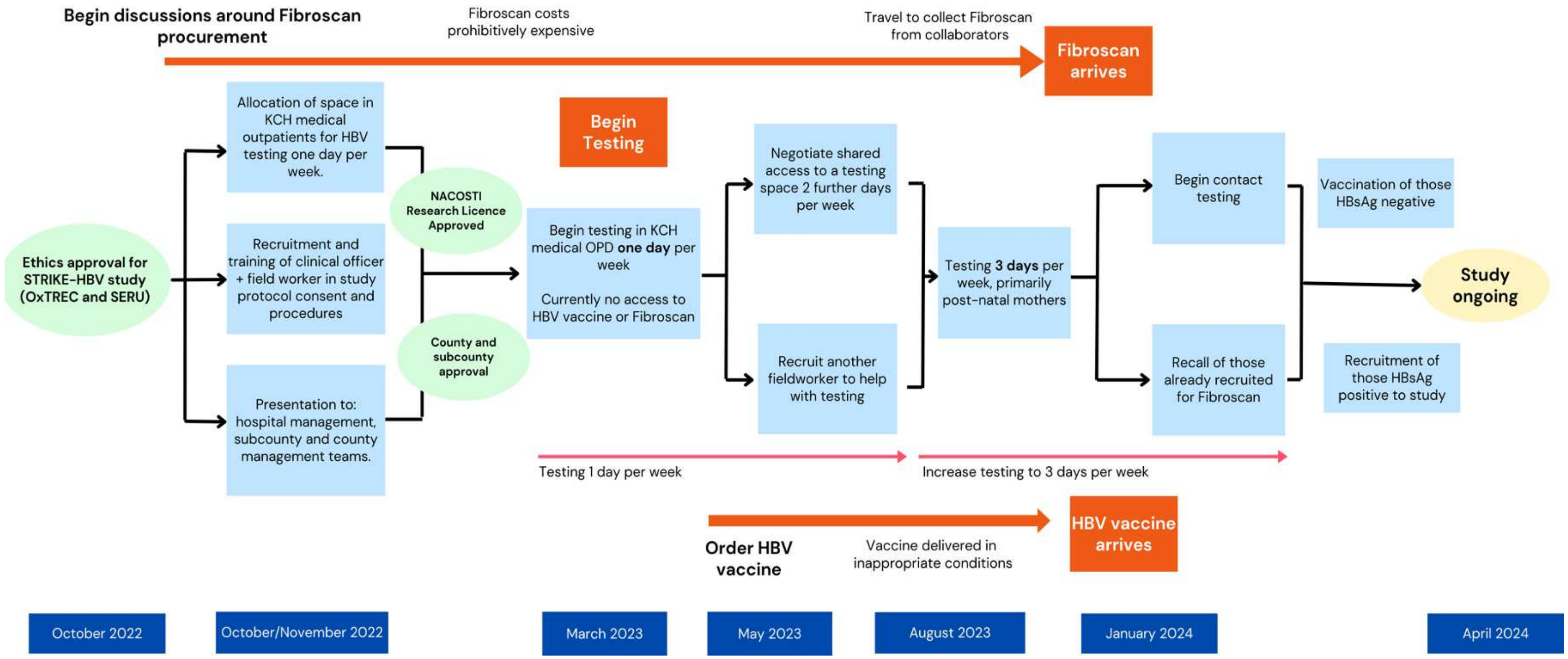

Supplementary figure 1: Flow diagram illustrating progression of the STRIKE-HBV study over time, a study screening people attending medical outpatient clinics in Kilifi County Hospital, Kenya for hepatitis B infection. HBV - Hepatitis B virus; KCH - Kilifi County Hospital - OxTREC Oxford Tropical Network Ethics Committee; SERU - Scientific Ethics Review Unit; NACOSTI - National Commission for Science Technology and Innovation; OPD – outpatients department; HBsAg – hepatitis B surface antigen.
